# Chiral Light–Matter Interaction for Controlling Nanoplasmonic Fields

**DOI:** 10.1002/nap2.70154

**Published:** 2026-06-03

**Authors:** Lázár Tóth, Judit Budai, Zsuzsanna Márton, Péter Rácz, Péter Dombi, Zsuzsanna Pápa

**Affiliations:** ^1^ ELI ALPS The Extreme Light Infrastructure ERIC Szeged Hungary; ^2^ University of Szeged Szeged Hungary; ^3^ HUN‐REN Wigner Research Center for Physics Budapest Hungary

## Abstract

The dichroic response of plasmonic nanostructures shows great potential for controlling chiral light–matter interactions. This approach is also promising for all‐optical switching using low‐intensity optical beams. In this work, plasmonic circular dichroism is demonstrated using closely spaced gold nanoparticle dimers, exhibiting high near‐field contrast under left‐circularly polarized (LCP) and right‐circularly polarized (RCP) illumination. Using scanning near‐field optical microscopy (SNOM), the dichroic behavior of the dimers under LCP and RCP illumination is mapped, enabling hot spots to become cold spots with full optical control. More importantly, the eigenmode excitations and their contributions to the plasmonic chiroptical response are directly revealed, which enables the complete understanding of the chiral behavior in complex nanostructures. These findings are valuable for designing nanophotonic devices.

## Graphical abstract

This paper demonstrates plasmonic circular dichroism using gold nanoparticle dimers that exhibit high near‐field contrast under left‐ and right‐circularly polarized illumination. SNOM mapping reveals that the dichroic behavior of the dimers transforms hot spots into cold spots with full optical control. Additionally, the eigenmode excitations and their contributions to the plasmonic chiroptical response are directly revealed.

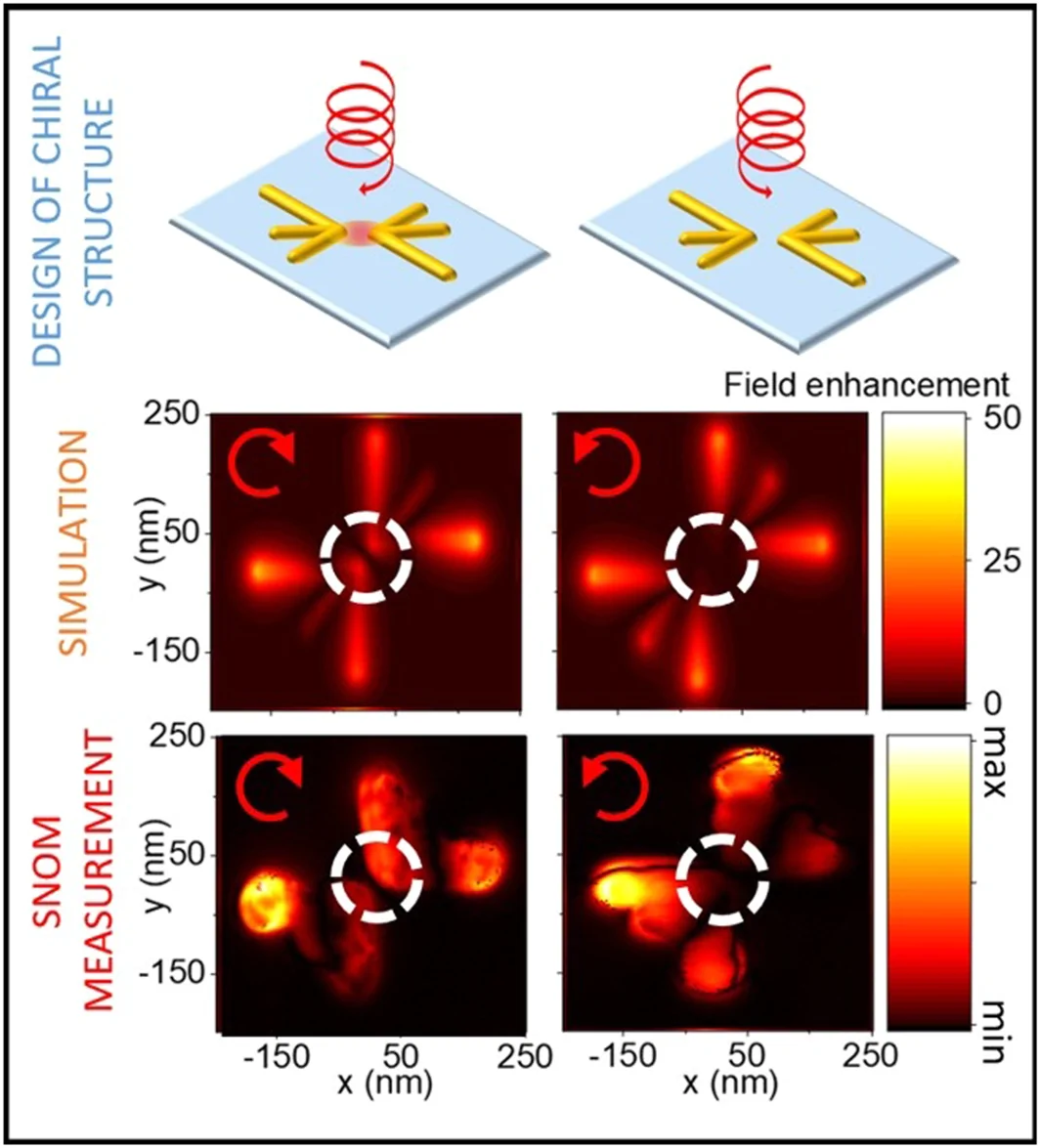

## 1 Introduction

Systems that lack mirror symmetry are very important building blocks of life including amino acids and sugars [1]. Molecular chirality is manifested by two nearly identical molecules having identical groups attached to a central atom that cannot be overlapped by rotation of the molecule. The optical analog of such mirror symmetry breaking is the application of circularly polarized light, where changing the helicity from left to right makes excitations non‐mirror symmetric [2]. From the point of view of interactions between circularly polarized light and molecules, there are two important aspects: (i) selective excitation of only one of the enantiomer pair by changing the helicity [3] and (ii) promotion of photoreactions in predefined pathways [4, 5]. Similar extinction properties for the two polarization states are also important from the point of view of energy dissipation.

Simply illuminating the molecules with light cannot provide either of the above aspects, as optical selectivity is usually poor due to the small difference in absorption coefficient between left‐ and right‐circularly polarized light [6], and there is no way to ensure spatial control in the subwavelength range [7]. Such criteria can be fulfilled by the application of a plasmonic metasurface.

Another field, where subwavelength spatial control of light fields is highly desirable, is all‐optical switching. All‐optical switching scheme means that a light beam controls another without the electronic conversion step. As such, ultrafast photonic devices have become available, opening the pathway toward efficient photonic information processing and all‐optical computing. All‐optical nanoswitches commonly rely on a nonlinear optical effect induced by the control beam [8, 9] or the excitation of hot‐electrons [10, 11]. These phenomena change the refractive index or absorption of the material, thereby modulating the transmission, reflection, or phase of a signal beam. However, the schemes mentioned suffer from high power requirements. Plasmonics can help here as well by exploiting surface plasmon resonances to concentrate light into nanoscale volumes, significantly enhancing light–matter interactions [12, 13, 14].

Localized surface plasmons (LSPs), which are collective oscillations of conduction electrons at metallic nanostructures, are widely used in experiments on light–matter interaction, due to their tunable properties depending on the desired application [15, 16, 17]. Specially designed plasmonic structures can even exhibit chiral behavior [18, 19, 20, 21, 22]. Interestingly, achiral structures can also generate circular dichroism as it is shown in refs [23, 24, 25, 26, 27]. Exploiting the field enhancement of a chiral plasmonic structure is one of the practical approaches to enhance the optical activity of molecules in the vicinity of the structure, leading to enhanced chiral catalysis or sensitive detection of biomolecules [5, 28, 29, 30, 31]. Such plasmonic structures also have the potential to actively manipulate the optical field within an extremely miniaturized spatiotemporal scale, paving the way for an all‐optical controlled modulator in a photonic circuit [32].

To achieve the active spatial control of the local field concentration (i.e., position of hot spots) and associated field enhancements, we exploited the interference of the plasmonic modes [33, 34]. Our concept is based on an arrangement of plasmonic nanorods where the phase relations between the eigenmodes mimic the phase between the orthogonal components of circularly polarized light. This way right‐circularly polarized (RCP) illumination can result in constructive interference of the eigenmodes leading to enhanced near‐fields, while left‐circularly polarized (LCP) excite the eigenmodes in opposite phase, resulting in a cold spot. As such, they are able to actively manipulate the field distribution by exploiting local interferences. For our investigations, we used 1550 nm light, but the concept can be easily adapted to other wavelength ranges.

### 1.1 Nanooptical Design for Polarization Control of Near‐Fields

As shown in our previous study, tuning the length of plasmonic nanorods makes it possible to adjust the relative phase of the dipolar plasmonic mode (along one axis of the rod that aligns with the direction of the linearly polarized illumination) relative to the phase of the exciting light wave [35]. In analogy with the classical oscillator model, a phase jump of π occurs around the spectral position of localized surface plasmon resonance. Thus, we can easily set an arbitrary phase difference between the nanorod eigenmodes by tuning their length. These nanorods can then be assembled into complex nanostructures with engineered phase relations for exhibiting circular dichroism.

For a circularly polarized light wave, the two orthogonal electric field component vectors are of equal magnitude and their phase difference is π/2. Thus, we can perfectly match our nanostructure to this polarization state if we assemble two nanorods of different lengths to have π/2 phase difference between their modes and place them orthogonally (see Figure 1a). Even higher field enhancement contrast is expected if a third nanorod is placed in 45° orientation with phase difference of π/4 (Figure 1b). A dimer of such a complex nanostructure is expected to show high field enhancement in the gap, since the nanorods are excited perfectly in‐phase with circularly polarized light. At the same time, by changing the helicity from RCP to LCP or vice versa, a high dichroic contrast is foreseeable due to the in‐phase and out‐of‐phase excitation of the dipolar modes (further details about the role of the constituting nanorods are discussed in the Supporting Information S1).

**FIGURE 1:**
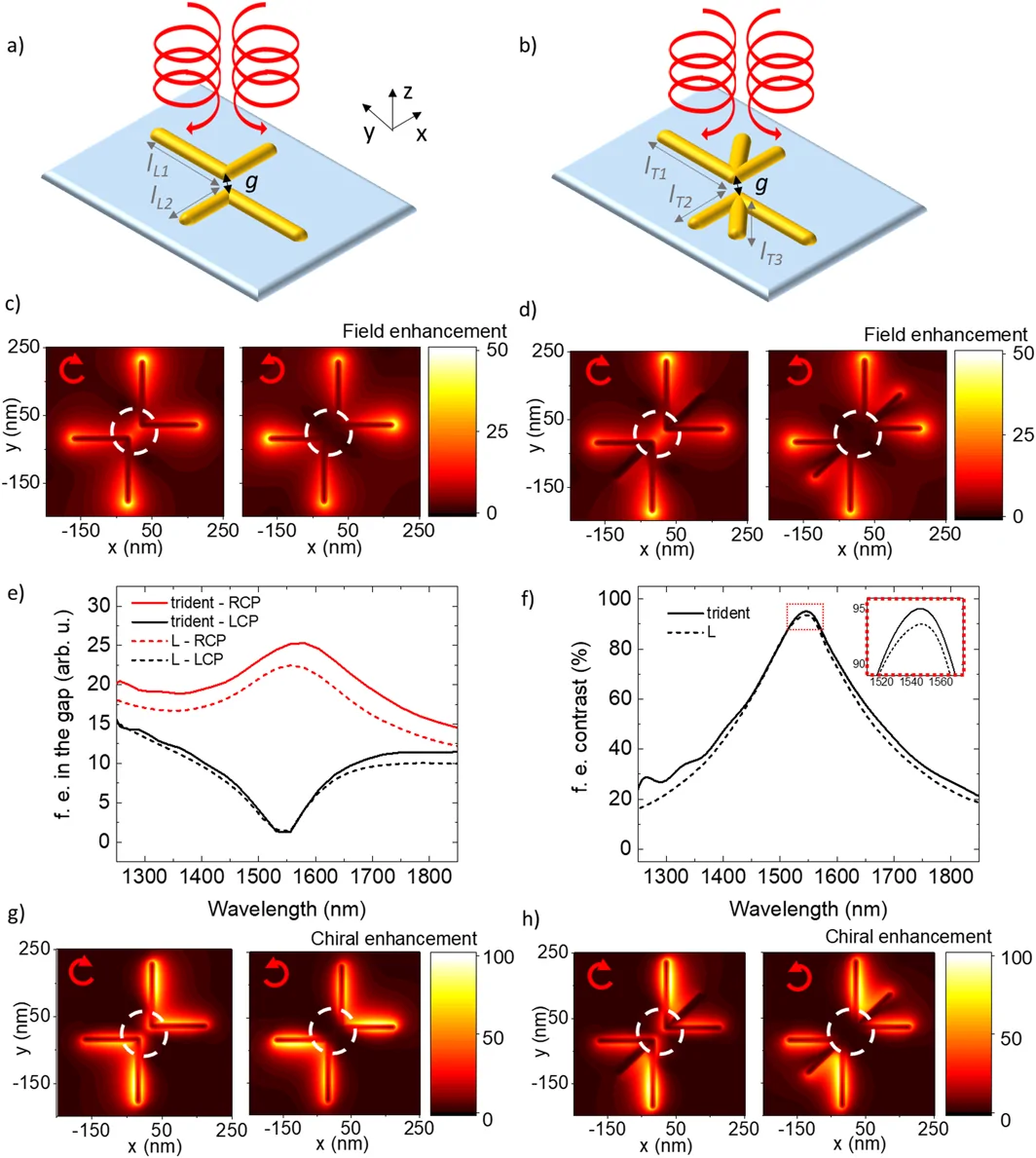
(a, b) Schematic illustration of the proposed nanostructure dimers: (a) L‐shaped structure, (b) trident structure. (c, d) Near field distribution of the two nanostructure types exhibiting the largest dichroic contrast in the gap (*l*_L1_ = 225 nm and *l*_L2_ = 190 nm, and *l*_T1_ = 240 nm, *l*_T2_ = 195 nm and *l*_T3_ = 180 nm) illuminated with right‐ and left‐circularly polarized light of 1550 nm wavelength. The color bar shows the field enhancement factor. White circles show the region where the highest differences are expected. (e) Field enhancement factor in the hot spot of the dimer's gap as a function of the excitation wavelength. (f) Field enhancement contrast in the gap, as defined by Equation (1). Inset: enlarged image at the wavelength range of maximal contrast. (g, h) Chiral enhancement distributions of the two nanostructure types.

To test this concept, we simulated nanorods with different length values and same width, and arranged them into the L‐shaped, and trident dimer structures as shown in Figure 1a,b, and calculated their field enhancement factor at the gap (defined as the field strength of the near‐field in the vicinity of the metamaterial surface normalized to the far‐field, *Q* = *E*_near_/*E*_far_) with finite difference time domain (FDTD) method (ANSYS Lumerical FDTD Solutions, see Supporting Information S1 for simulation details). For the simulations, we used RCP and LCP excitations. We defined the following quantity to represent the dichroic response (*D*) and quantize the contrast in percentage:

(1) $$D=100\cdot \left(1-\frac{Q_{\text{LCP}}}{Q_{\text{RCP}}}\right),$$

where *Q*_LCP_ is the maximal field enhancement at the gap calculated using LCP illumination, while *Q*_RCP_ is the same for RCP. Values of *D* range from 0 to 100, with higher values indicating greater dichroic contrast. We tested which combination of lengths of the constituent nanorods results in the highest *D* values. During optimization, we performed several FDTD simulations, varying the size of each leg by 5 nm. The width of the nanorods forming the complex structures is 40 nm in all cases. The highest *D* value belongs to the L‐shaped dimer having *l*_L1_ = 225 nm and *l*_L2_ = 190 nm long legs, and for the trident particle dimer, the optimal length values are *l*_T1_ = 240 nm, *l*_T2_ = 195 nm and *l*_T3_ = 180 nm. Besides the high contrast, the optimized structures indeed show a prominent field concentration in the gap for RCP (Figure 1c,d). This field concentration in the gap region can almost perfectly be suppressed by changing the helicity, ensuring the desired dichroic response. The contrast of the achieved field enhancement factors in the gap with LCP and RCP light is almost 95% for the trident structure, which means that the hot spot in the nanogap can be turned into a cold spot with our circularly dichroic sample concept (Figure 1e). The FWHM value of this dichroic contrast is > 250 nm, meaning that our proposed structure exhibits dichroic response in a large bandwidth (Figure 1f).

We have to note that for effective chiral detection, field enhancement factor is just one ingredient. High field enhancement itself is not sufficient to obtain a strong optical chirality as the polarization of the incident light is not guaranteed to be maintained by the field‐enhancement effect. Therefore, the chiral enhancement factor should also be considered when chiral detection is aimed. *K* is defined in terms of the optical chirality C,

(2) $$C=\frac{\varepsilon_{0}\omega }{2}Im\left[E\cdot H^{*}\right],$$

such that $K=C_{\text{wi}}/C_{\text{wo}}$, where $C_{\text{wi}}$ is the optical chirality in the vicinity of the nanostructures illuminated with circularly polarized light, while $C_{\text{wo}}$ corresponds to the chirality recorded at the same position without the nanostructures. This definition provides a direct physical interpretation, where K quantifies the enhancement of the local chiral electromagnetic field responsible for enantioselective light–matter interactions [18].

Importantly, for a chiral molecule, the differential absorption between left‐ and right‐circularly polarized light scales as ΔA∝C, where the proportionality constant depends on the properties of the molecules. Therefore, the enhancement factor K directly translates into an enhancement of the measurable chiral signal, that is, $\Delta A_{\text{wi}}\approx K\cdot \Delta A_{\text{wo}},$ where $\Delta A_{\text{wi}}$ and $\Delta A_{\text{wo}}$ are the differential absorption values with and without the nanostructures [36, 37]. Our simulations show that—similar to the near‐field enhancement factor—the optical chirality in the gap of nanostructure dimers can also be switched with circularly polarized light (see Figure 1g,h for the L‐shaped and trident structures, respectively). Under right circularly polarized illumination, the gap region exhibits strong field confinement (*Q* > 25) and large chiral enhancement (*K* > 40), resulting in a pronounced chiral hotspot. In contrast, under left circular polarization, both quantities are suppressed, leading to a negligible chiral response. This polarization‐selective behavior demonstrates the proposed system’s potential for highly sensitive detection of chiral molecules.

Table S1 of the Supporting Information S1 provides a comparison with some examples from the literature, including field and chiral enhancement factors.

## 2 Characterization of the Nanoantenna Array

To test the dichroic behavior of L‐ and trident structures experimentally, the optimized nanostructures were fabricated on quartz plates using electron beam lithography (Raith e‐LINE Plus). The structure sizes were characterized by scanning electron microscopy (insets of Figure 2). Plasmonic extinction spectra were measured by standard near‐infrared (< 1650 nm) extinction measurements using a ZEISS AXIO Observer 3 microscope in transmission mode and an Ocean Optics Flame NIR spectrometer.

**FIGURE 2:**
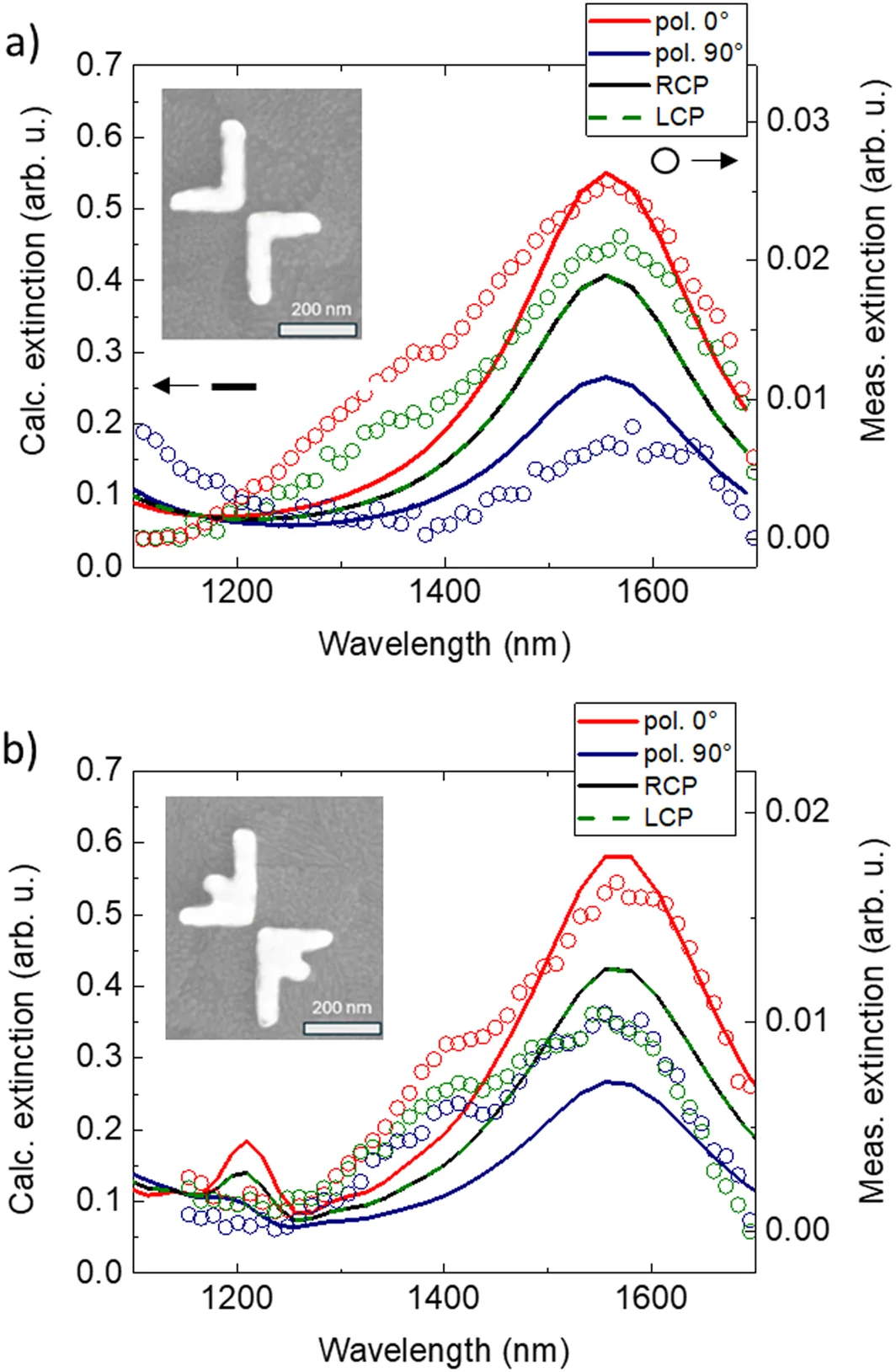
(a, b) Measured and simulated extinction spectra of (a) the L‐shaped and (b) trident structures with linearly polarized excitation along the main axes of the nanostructures (0° and 90° orientation) and circular polarization. Insets: SEM images of the L‐shaped and trident dimer structures.

Figure 2a,b show the simulated and measured extinction spectra of the nanostructure dimers. The resonance position is the same for the two orthogonal linear polarizations (0° along *x*‐axis and 90° along *y*‐axis; for further explanation, see Supporting Information S1), and more importantly, there is no difference between the spectra belonging to RCP and LCP illumination. This confirms that the main origin of the dichroic effect is the phase difference between the plasmonic eigenmodes, which cannot be visualized by an extinction measurement. Additionally, this meets the criterion of having no significant difference in power dissipation between the RCP and LCP illumination schemes.

In addition to the nanostructures shown in the SEM images, we also fabricated their chiral counterparts, that is, the mirrored version of them. This allowed us not only to compare the responses of the same structure under RCP and LCP illumination, but also to compare the chiral nanostructure counterparts under the same illumination conditions.

## 3 Local Field Probing With SNOM

Although absolute field enhancement values are routinely available with the help of ultrafast photoemission [38, 39, 40, 41, 42, 43], in this case, we needed local field mapping to validate our concept. Therefore, we implemented a scanning probe method.

To directly demonstrate plasmonic circular dichroism, we used a scanning near‐field optical microscope (Neaspec s‐SNOM), where the desired RCP and LCP polarization states of the 1550 nm CW illumination were adjusted with a half‐wave‐plate and a quarter‐wave‐plate (see Supporting Information S1). This technique allows us to image both magnitude and phase of the near field simultaneously. Due to the elongation of the SNOM tip along the *z*‐axis, the recorded near‐field signal corresponds well to the normal *z*‐component of the electric field.

The measured signal in a SNOM device is affected not only by the sample properties and the incident illumination, but also by the dielectric properties of the tip, its average height, radius and tapping amplitude (Δ*z*) [44]. Choosing a small amplitude suppresses background scattering more efficiently and confines the near‐field signals in the *z*‐direction [45], but the signal is noisier in this case. Higher amplitudes increase the signal‐to‐noise ratio, but are more likely to degrade the tip and enhance the contribution of the background. For our measurements, we chose an average tapping amplitude of 60–70 nm as a compromise.

Typical measured amplitude maps are shown in Figure 3 for the optimized L‐shaped and trident structures along with the simulated distribution of the *E*_*z*_ field component. The simulated maps were taken 40 nm above the nanostructure similarly to the method described in [46]. It can be clearly seen without any further evaluation that there is a large difference between the RCP and LCP illumination when the gap region is considered. For RCP, a high field amplitude occurs in the gap, while for LCP the fields are almost perfectly suppressed.

**FIGURE 3:**
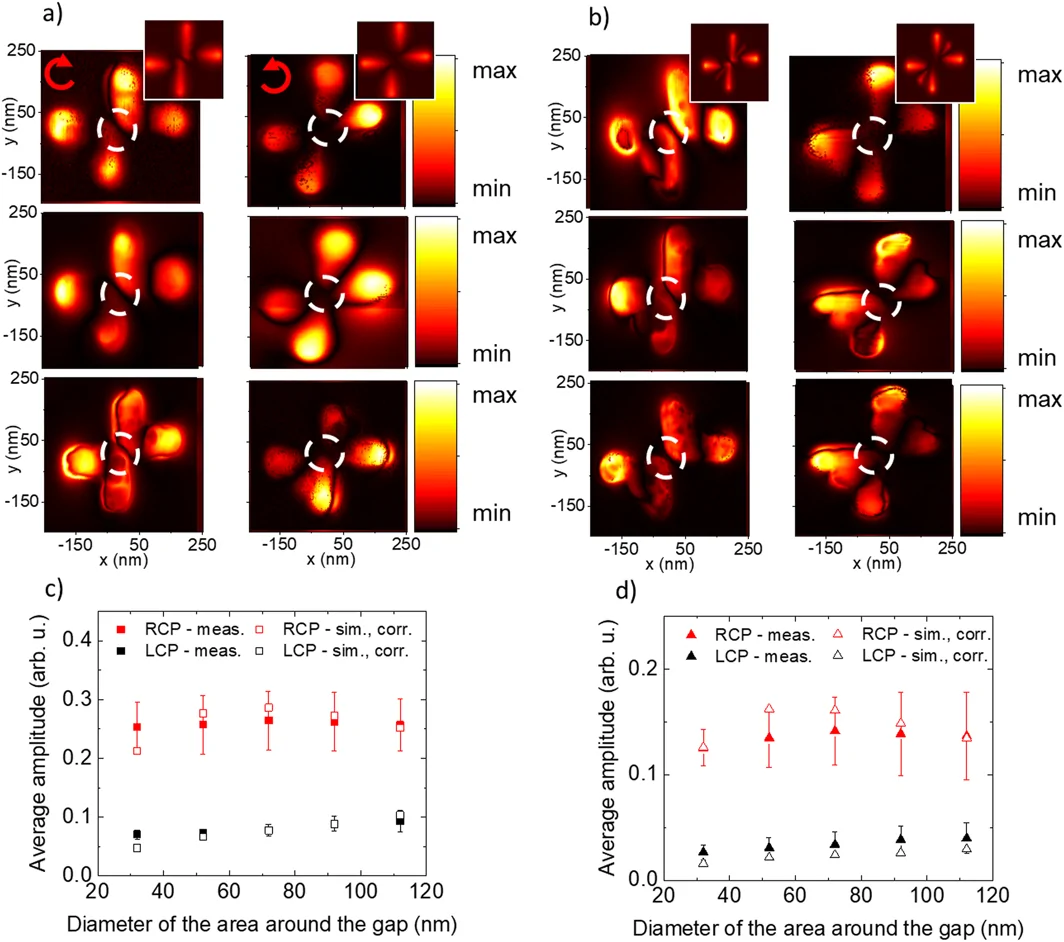
(a) Measured amplitude maps of the optimized L‐shaped structure with RCP and LPC illumination at 1550 nm. (b) Same images for the trident structure. The insets show the simulated distribution of the *Ez* field component recorded 40 nm above the structure. (c, d) Amplitude values averaged for different areas around the gap. Error bars are from the analysis of several measurements.

To quantify these differences, we calculated the average amplitude within circular regions of different sizes centered in the middle of the gap. This allows us to determine the size of the region where the circularly dichroic response is present. Figure 3c,d show these averaged amplitudes from the measured and simulated amplitude maps. The error bars for the measurements come from the evaluation of amplitude maps recorded on 3–3 adjacent nanostructures and their chiral counterparts. Below 100 nm diameter averaging, the LCP amplitudes are very low, confirming that the cold spot in the center extends to a significant size compared to the nanostructure size. The trends of the averaged measured amplitudes for the different illuminations are in good agreement with the simulation results. (Note that the simulated amplitude values are corrected with a scaling factor for better comparison).

Further information about the performance of our fabricated nanostructures can be obtained by evaluating the field enhancement contrast (see the dataset recorded with Δ*z* = 66 nm tapping amplitude in Figure 4a, based on Equation (1)). For the Δ*z* = 66 nm dataset of Figure 4a, the error bars come from the evaluation of field enhancement contrast on 3–3 adjacent nanostructures and their chiral counterparts. Note that the measured contrast is much smaller than the contrast of the hot spots used for optimization (> 90%, see Figure 1f). The reason for this is twofold. During optimization, the hot spot amplitudes were compared, while for the evaluation, we used average amplitudes. In addition, the amplitude and contrast of the SNOM signal is partially determined by how close the tip can approach the surface. In our simulations, data recorded a few tens of nanometers above the surface are used (*z* = 40 nm) to support measurements. Taking into account the decay of the near‐fields, the contrast also decreases as we move away from the plane of the sample surface (see Figure 4b). At a distance of 40 nm, even the hot spot contrast drops to 80% compared to the value of 95% at the surface.

**FIGURE 4:**
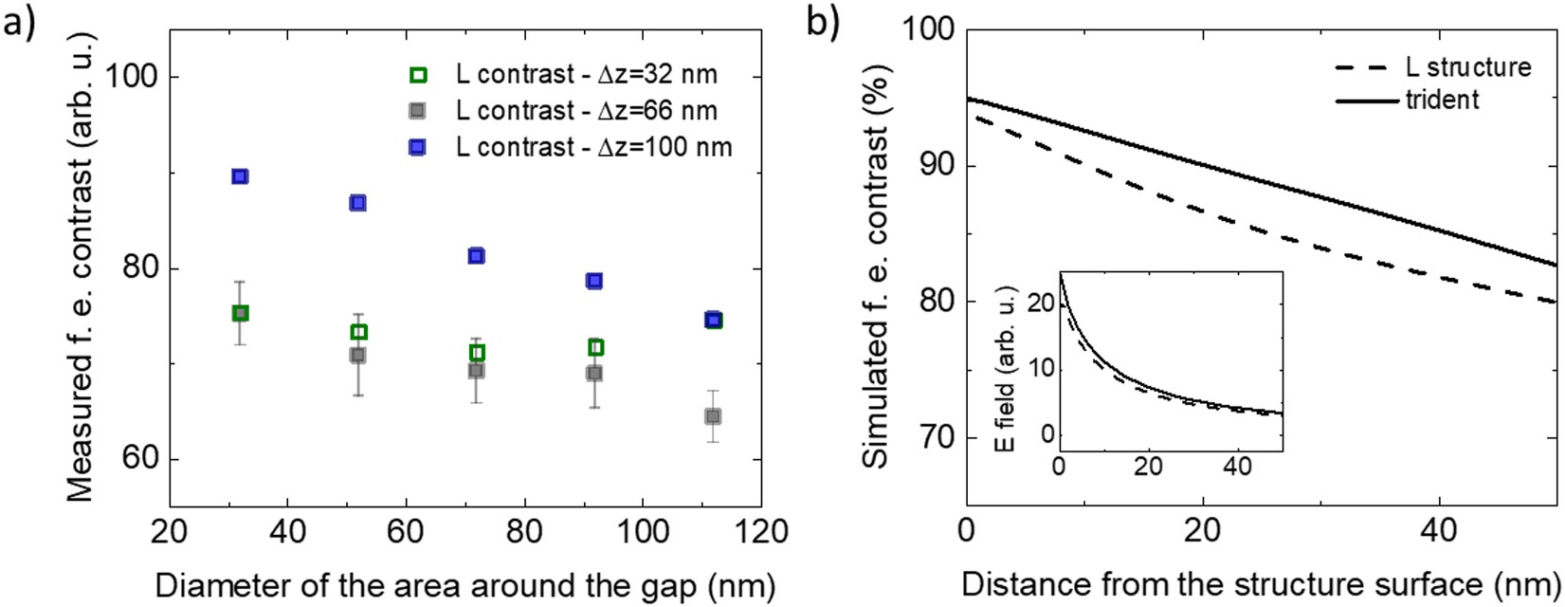
(a) Measured field enhancement contrast of the optimized L‐shaped structure with different tapping amplitudes (Δ*z*). (b) Simulated field enhancement contrast of the L‐shaped and trident nanostructures at 1550 nm as a function of the distance from the sample surface. Inset: Spatial decay of the electric field for RCP illumination.

To analyze this effect in more detail, we recorded SNOM amplitude maps from the same nanostructures with different tapping amplitudes, and calculated the field enhancement contrast for these measurements, as well (blue and green symbols in Figure 4a). As the tapping amplitude increases, the sample‐tip interaction becomes stronger, resulting in closer contact [45]. Consistent with the simulated distance dependence of the contrast (Figure 4b), the measurements with the highest applied Δ*z* value exhibit the highest contrast values. This indicates that the samples indeed exhibit a strong circularly dichroic response, although full quantitative agreement between the optimization and SNOM measurements is difficult to achieve.

## 4 Conclusions

We proposed a concept for designing nanostructures exhibiting ultrahigh plasmonic circular dichroism based on the local interferences of plasmonic modes. Our simulations and SNOM experiments verified the dichroic behavior by providing almost 95% field enhancement contrast in the gap by using LCP and RCP illuminations, respectively. We also identified the size of the gap region where the hot‐to‐cold spot switching is best fulfilled.

The concept of polarization sensitivity of plasmonic mode excitation has already found applications in all‐optical switching [11, 14, 34]. Complementing these results with the possibility of tuning the plasmonic modes to the excitation can be further exploited in diverse designs of local field responses with the desired, unique spatial distributions and temporal dynamics, expanding the possibilities of nanospectroscopy and nanooptical control. Therefore, our study opens a pathway toward better plasmonic engineering and ultrafast, surface‐integrated switching architectures [10].

## Author Contributions

**Lázár Tóth:** conceptualization, investigation, writing – original draft, visualization. **Judit Budai:** writing – review and editing, validation, methodology. **Zsuzsanna Márton:** methodology, validation, software. **Péter Rácz:** conceptualization, writing – review and editing. **Péter Dombi:** conceptualization, supervision, resources. **Zsuzsanna Pápa:** conceptualization, writing – original draft, supervision, validation, visualization.

## Conflicts of Interest

The authors declare no conflicts of interest.

## Supporting information


Supporting Information S1

## Data Availability

The data that support the findings of this study are available from the corresponding author upon reasonable request.
