# Supplementary material for: Chiral Light–Matter Interaction for Controlling Nanoplasmonic Fields
Source: Nanophotonics. 2026 Jun 3;15(11):e70154. doi: 10.1002/nap2.70154 (PMC13642055; doi:10.1002/nap2.70154)
Supplement: Supplementary file 1 [file NAP2-15-e70154-s001.docx]

Supporting information

**Chiral light-matter interaction for controlling nanoplasmonic fields**

Lázár Tóth, Judit Budai, Zsuzsanna Márton, Péter Rácz, Péter Dombi*, Zsuzsanna Pápa*

**1. Details of FDTD simulations**

The near-field properties around the nanostructures were calculated using a commercial finite-difference time-domain (FDTD) solver (ANSYS Lumerical FDTD solutions). The simulations included a 3D unit cell with perfectly matched layer (PML) boundaries. The applied thickness for gold was 40 nm on a bulk fused silica substrate. The refractive index of gold was taken from the literature [1], while for fused silica n=1.45 was used. According to SEM images, the gold nanostructures had rounded edges and the radius of curvature was set to 22 nm.

A total-field scattered-field (TFSF) source with a center wavelength of 1550 nm was used to simulate the plane-wave excitation of the nanostructure. The circular polarization was adjusted by using two identical linearly polarized sources with different phase characteristics. To determine the extinction spectrum of the nanostructures, the absorbed and scattered power was recorded using frequency domain field monitors. The near-field distributions around the nanostructures were recorded with the same monitor types in the plane of the structure surface and 40 nm above. All simulations were preceded by a rigorous convergence check.

**2. Fabrication of the nanostructure array with electron beam lithography**

The optimized nanostructures were fabricated onto quartz plates with electron beam lithography (Raith e-LINE Plus). Before the EBL process, the cleaned quartz substrates were coated with the EBL resist (Allresist AR-P 672.03 PMMA (poly-methyl methacrylate) positive tone resist) via spin coating (2500 rpm), and then the sample was shortly annealed using a hotplate at 155°C. For the electron irradiation, we used 25 kV voltage, 7.5 µm aperture and 180 µC/cm^2^ dose. These samples were developed for 45 seconds (Allresist 600.55 developer) to remove the irradiated parts, and in isopropyl-alcohol for 60 s to stop this process. 2 nm Cr and 40 nm Au were evaporated on the sample surface in a vacuum chamber (pressure below 10^-6^ mBar). The last step was the lift-off in acethone bath.

To obtain information about size variations of the nanostructures made during our EBL process, we analyzed rectangular nanoparticles comparable in size to our L-shaped and trident nanostructures and plotted their size distribution in a histogram (Fig. S1 a)). This analysis resulted in a ±6 nm size variation. Next, we examined how these inaccuracies affect the field enhancement contrast of our nanostructure dimers in the gap. Based on FDTD results, we found that fabrication inaccuracies cause a contrast decrease of less than 20%.

**Figure S1 a)** Length distribution of plasmonic nanorods fabricated with EBL. b) Field enhancement contrast in the gap of L dimer structures of varying sizes. Black rectangle shows the size variation range.

It has to be noted that EBL is not a suitable method for mass production of such samples. However, once the design is established, the fabricated structures can serve as master templates for scalable replication techniques such as nanoimprint lithography and soft lithography. These methods are widely used to reproduce plasmonic metasurfaces and nanostructured patterns over wafer-scale areas with high throughput and significantly lower cost [2,3].

**3. SNOM measurements**

In our Neaspec s-SNOM device a 1550 nm cw laser was used in transmission mode with 12 mW power. During each measurement, a 1x1 µm^2^ area was scanned with 4 nm/pixel resolution and 0.4 µm/s scan speed. The typical tapping amplitude of the AFM tip (Nanoworld Arrow NcPt Non-Contact mode sensor with 20 nm radius) was 60-70 nm.

**Figure S2.** Upper row: Measured amplitude and phase maps of a nanodisk illuminated with linear 45° polarized light. Insets: simulation results under the same conditions. Lower row: Same for circular polarization.

The desired right and left circularly polarized illumination in the SNOM system was adjusted with a half-wave plate and a quarter-wave plate. To verify the set polarization states, we scanned a sample containing gold nanodisks, since the field distribution of such nanodisks is very sensitive to polarization, and shows significant differences when linear or circular polarization states are set. Measurements were performed in two cases. The top row of Fig. S2 shows the amplitude and phase measurements made in the default polarization state of the SNOM (linear 45°). The lower figures show measurements with a half-wave plate and a quarter-wave plate inserted to produce circularly polarized light. There is a slight asymmetry in the amplitude map due to the imperfect surface. The simulated (insets) and the measured disks look similar in both cases, verifying the set polarization states.

**4. Behavior of L-shaped nanostructures**

In designing the L-shaped and trident nanostructures, we first analyzed the phase relations of single nanorods. Since the resonant nanorods have a length of ~300 nm at 1550 nm, we investigated nanorods with lengths between 175 and 475 nm. We then plotted their relative phases compared to the phase of the exciting laser pulse (Fig. S3).

**Figure S3.** LSP phase relative to the excitation corresponding to the plasmonic modes for 40 nm wide nanorods having different lengths for 1550 nm excitation wavelength.

Considering the phase differences shown in Fig. S2, the nanorods forming the optimized L-shaped and trident structures with length values between 180 nm and 240 nm should not exhibit the desired π/2 phase differences at 1550 nm excitation. To explain why these nanorods will still provide the required phase differences we have to consider the more complex resonance landscape of closely spaced / touching nanorods. Fig. S3a shows the resonance properties of a 225 nm long nanorod, and three L-shaped structures consisting of a 225 nm long nanorod (horizontal leg of the L) along the direction of linearly polarized excitation (red arrow in Fig. S3a), and another nanorod of increasing length in the orthogonal direction (vertical leg of the L form). Although the length of the structure along the polarization direction is the same, the presence of the vertical leg shifts the resonance to longer wavelengths. To analyze this behavior, we calculated the current density of the L-shaped structures according to the following formula:

$J=-i\omega\left( \varepsilon-\varepsilon_{0} \right)E$	(S1)

where J is the surface current density, *ω* is the angular frequency, *ε* and *ε_0_* are the permittivities of the material and the environment (air), and E is the electric field calculated from FDTD simulations. Fig. S2b shows that in L-shaped structures, the charge separation along the polarization direction also leads to an instantaneous surface current along the vertical leg (see Fig. S4b for the 225 nm x 150 nm L-structure) [4], and the excitation of the corresponding plasmonic mode is coupled to the horizontal one. The overall behavior of such resonance will be very similar to that of a longer nanorod (see the comparison of 225 nm x 150 nm L structure and 350 nm long nanorod in Fig. S4a). Thus, the L-structures formed by shorter rods can indeed exhibit the desired phase properties.

**Figure S4.** a) Calculated resonance of a nanorod and three L-shaped structures of equal length along the polarization direction, with increasing orthogonal leg length. The dashed line shows the resonance of a 350 nm long nanorod being almost equal to the resonance of the 225 nm x 150 nm L-shaped structure. b) Electric field induced current density distribution in the 225 nm x 150 nm L-shaped structure. The polarization of the illumination is indicated by the red arrows.

**4. Role of nanorods in the dimer nanostructures**

To gain a deeper understanding of the role of nanorods in forming L-shaped and trident structures, we analyzed the amplitude and phase of the E_z_ component for different structures (see Figs. S5 and S6). The E_z_ distributions clearly show that, for the L-shaped structures, the sign of the E_z_ changes with helicity. For the trident structures, however, the E_z_ distribution remains almost unchanged except in the gap region. This is due to the excitation of different modes. As expected, the L-dimer structure supports two dipolar modes that are orthogonal to each other and are associated with its legs. Since the legs have different lengths, the modes exhibit different resonances, resulting in a distinct phase difference between them at 1550 nm. These phase properties enable in-phase superposition of the modes for left-circular polarization and out-of-phase superposition for right-circular polarization (or vice versa). As such, the gap region will exhibit large field enhancement due to constructive or almost no fields for destructive interferences. At the same time, the sign of the phase distribution flips with helicity.

**Figure S5.** Near field distribution of the orthogonal electric field component of the two nanostructure types illuminated with right- and left-circularly polarized light of 1550 nm wavelength. Color bars show the normalized value of E_z_.

In contrast, a trident geometry introduces an additional 45° leg, which increases the in-plane rotational symmetry. Thus, the system supports three coupled dipoles and their combinations, including rotating modes that resemble angular momentum states [5]. Circular polarization then couples more directly to these rotating modes. Consequently, although the behavior of the gap region is sensitive to helicity, the spatial distribution and phase of the z-component of the electric field remain essentially unchanged. This behavior is consistent with the plasmon hybridization framework [6].

**Figure S6.** Phase distribution of the orthogonal electric field component of the two nanostructure types illuminated with right- and left-circularly polarized light of 1550 nm wavelength. Top row: measured phase maps, bottom row: simulated phase maps. Color bars show phase values in rad. Dashed lines highlight important features.

**5. Comparison with literature results**

Table S1 provides an overview of existing chiral nanoparticle platforms based on relevant literature results.

|  | Fabrication Method | Field Enhancement | Chiral Enhancement (CD / g-factor or local) | Bandwidth | Polarization Selectivity |
| --- | --- | --- | --- | --- | --- |
| This work: Chiral nanoparticle dimers | Electron beam lithography (EBL) | ~25× (gap hotspot) | ~40× (local chiral field) | Narrow–moderate (plasmonic resonance) | Strong (LCP suppressed) |
| Twisted metamaterials (e.g., [7]) | Multilayer lithography / self-assembly | ~10–20× | ~5–15× (far-field CD) | Broadband | Moderate (both polarizations interact) |
| Core shell (e.g., [8]) | Colloidal synthesis | ~5–15× | ~10–30× | Moderate | Weak–moderate |
| Helical plasmonic structures [9] | 3D nanofabrication | not emphasized | ~10–20× | Narrow–moderate | Strong (intrinsic chirality) |
| Planar nanostructures (gammadiona) [9] | EBL | not emphasized | ~20–30× | Narrow–moderate | Strong (intrinsic chirality) |
| 3D nanostructures [9] | layer-by-layer lithography | not emphasized | ~100× | Narrow–moderate | Strong (intrinsic chirality) |
| Dielectric nanostructures [10] | EBL | ~10× | ~50× | Broadband | Strong |

**Table S1.** Performance comparison of existing chiral nanoparticle platforms.

References

1. Haynes, W. M., Ed., “CRC Handbook of Chemistry and Physics”, 96th ed.; *CRC Press* Taylor and Francis Group: Boca Raton, FL, 2015.
2. Wang, P., Krasavin, A. V., Liu, L., Jiang, Y., Li, Z., Guo, X., Tong, L., Zayats, A. V., “Molecular Plasmonics with Metamaterials.” *Chem. Rev.* 122, 19, 15031–15081 (2022).
3. Malyarchuk, V., Hua, F., Mack, N. H., Velasquez, V. T., White, J. O., Nuzzo, R. G., Rogers, J. A., “High performance plasmonic crystal sensor formed by soft nanoimprint lithography.” *Opt. Express* 13, 5669-5675 (2005).
4. Jing, Y., Jia-Sen, Z., Xiao-Fei, W. & Qi-Huang, G., “Resonant Modes of L-Shaped Gold Nanoparticles.” *Chinese Physics Letters* 26, 067802 (2009).
5. Prinz, E., Hartelt, M., Spektor, G., Orenstein, M., Aeschlimann, M., “Orbital Angular Momentum in Nanoplasmonic Vortices.” *ACS Photonics* 10 (2), 340-367 (2023).
6. Nordlander, P., Oubre, C., Prodan, E., Li, K., Stockman, M. I., “Plasmon Hybridization in Nanoparticle Dimers.” *Nano Letters* 4 (5), 899-903 (2004).
7. Li, H., Ye, W., Polavarapu, L., Xie, J., Wong, K.-Y., Zheng, G., “Chiral plasmonic superlattice resonance based on metasurfaces for chiral molecular sensors.” *Opt. Lett.* 50, 2900-2903 (2025).
8. Luo, T., Li, H., Zhang, Z., Wang,S., Pan, X., Mourdikoudis, S., Xue, C., Li, J., Wong,K.-Y., Zheng, G., “Super-Heterostructures of Twisted Pd Nanoarrays Epitaxially Grown on Chiral Au Nanorods Boost Circularly Polarized Photocatalysis.” *Advanced Science* 12 2502848 (2025).
9. Schäferling, M., Dregely, D., Hentschel, M., Giessen, H., “Tailoring Enhanced Optical Chirality: Design Principles for Chiral Plasmonic Nanostructures.” *Phys. Rev. X* 2, 031010 (2012)
10. Mohammadi, E., Tavakoli, A., Dehkhoda, P., Jahani, Y., Tsakmakidis, K. L., Tittl, A., Altug, H., “Accessible Superchiral Near-Fields Driven by Tailored Electric and Magnetic Resonances in All-Dielectric Nanostructures.” *ACS Photonics* 6, 8, 1939–1946 (2019).
